# Risk factors for addictive disorders in life history interviews

**DOI:** 10.1192/j.eurpsy.2023.1187

**Published:** 2023-07-19

**Authors:** M. Krupa, E. Kiss, K. Kapornai

**Affiliations:** 1 Doctoral School of Education; 2 Child and Adolescent Psychiatry, University of Szeged, Szeged, Hungary

## Abstract

**Introduction:**

There are multifactorial pathologies in the development of addictive disorders, such as psychosocial factors, genetic and biological factors, as well as their interaction. In line with this, psychological research focuses on the abusive environment and its impact, where the developmental psychopathological analysis of addictive disorders is of paramount importance, since it examines the causes of the disease with the involvement of several disciplines.

**Objectives:**

In our research, we studied the risk factors leading to the development of addictive disorders through the lifetime histories of those who recovered from this disorder. Our goal was to point to the common factors that emerged in the interview narratives in the development of addictions. Furthermore, we revealed risk factors that affected the psychological processes influencing both personal and social functioning.

**Methods:**

We processed semi-structured interview materials from 12 adult patients who were previously treated for addictive disorder but were substance-free for more than 4 years. Distinguishable phenomena with guided questions emerged: peak experiences, lows, turning points, and first psychoactive substance use. The interviews distinguished childhood, adolescence and adulthood, as well as the best and worst substance use experiences.

**Results:**

Emotion regulation difficulties and low self-esteem emerged as dysfunctionality in most of the interviews. Without exception, the good effects of substance use appeared in the life stories, and led to the development of addictive disorder. In retrospective narratives, it is decisive and points toward recovery from the bad effects of the drug decisive presence. The narratives showed a change in the overall pattern, when self-control, performance, empowerment appeared. The road to recovery in the narratives led from illness to the pursuit of good emotion regulation and the strengthening of self-esteem.

A common narrative thread mostly showed a V-shape, which, unlike previous models, is a dynamic model. This new finding sheds light on the possibility of a recovery-centered model in adult population with addictive disorder.

**Conclusions:**

Disturbances in emotion regulation and low self-esteem could be experienced as early as in adolescence, correctly recognizing the risk factors of addiction. Therefore, prevention can be applied. In addition to the qualitative studies, it is also necessary to measure the risk factors by quantitative method, which can confirm the results.

We need to be mindful of the different characteristics of disease- and recovery-oriented narratives, which may differ due to various life history experiences.

**Disclosure of Interest:**

None Declared

